# European NSTEMI guidelines—return of clopidogrel?

**DOI:** 10.1007/s00228-021-03200-2

**Published:** 2021-09-15

**Authors:** Lisa Dannenberg, René M’Pembele, Daniel Metzen, Tobias Petzold, Tobias Zeus, Malte Kelm, Amin Polzin

**Affiliations:** 1 Medical Faculty, Division of Cardiology, Pulmonology, and Vascular Medicine, Cardiovascular Research Institute Düsseldorf (CARID), University Duesseldorf, Duesseldorf, Germany; 2 Department of Anesthesiology, University Hospital Duesseldorf, Duesseldorf, Germany; 3 Medizinische Klinik Und Poliklinik I, Klinikum Der Universität München, Ludwig-Maximilians-University Munich, Munich, Germany

**Keywords:** Academic Research Consortium, Clopidogrel, DAPT, High bleeding risk

Stent thrombosis and re-infarction are disastrous complications after acute myocardial infarction. During the last decade, potent P2Y12 inhibitors like prasugrel/ticagrelor and extended duration of dual antiplatelet therapy (DAPT) substantially reduced recurrent ischemic events. However, the novel European NSTEMI guidelines emphasize rigorous de-escalation of DAPT in patients with NSTEMI at “high bleeding risk” [1]. Definition of “high bleeding risk” is extremely challenging. In this study, we investigated how many patients would be de-escalated to 3 months of aspirin and clopidogrel according to the emphasized “high bleeding risk” algorithm in the ESC NSTEMI guidelines in a real-life cohort (Table 1).

**Table 1:** Rate of post PCI patients with non-ST-elevation myocardial infarction (NSTEMI) that fulfill major and minor criteria of the Academic Research Consortium (ARC) and the PRECISE-DAPT (PD) Score for high bleeding risk (HBR)

Academic Research Consortium (ARC) for high bleeding risk (HBR)			
**Major Criteria**	**No. (%)**	**Minor Criteria**	**No. (%)**
Anticipated use of long-term OAC	205 (21.1%)	Age ≥ 75 years	390 (40.0%)
Severe end stage CKD (eGFR < 30 ml/min)	69 (7.1%)	Moderate CKD (eGFR 30–59 ml/min)	293 (30.1%)
Hemoglobin < 11 g/dl	132 (13.6%)	Hemoglobin 11–12.9 g/dl for men or 11–11.9 g/dl for women	99 (10.2%)
Spontaneous bleeding requiring hospitalization and/or transfusion in the past 6 months or at any time if recurrent	2 (0.2%)	Spontaneous bleeding requiring hospitalization and/or transfusion in the past 12 months not meeting the major criterion	21 (2.2%)
Moderate or severe baseline thrombocytopenia (platelet count < 100 × 10^9^/L)	11 (1.1%)	Chronic use of non-steroidal anti-inflammatory drugs or steroids	58 (6.0%)
Chronic bleeding diathesis	2 (0.2%)	Any ischemic stroke at any time not meeting the major criterion	72 (7.4%)
Liver cirrhosis with portal hypertension	13 (1.3%)		
Active malignancy (excluding non-melanoma skin cancer) within past 12 months	0(0%)		
Previous spontaneous intracranial hemorrhage (at any time)	0(0%)		
Previous traumatic intracranial hemorrhage within past 12 months	0 (0%)		
Presence of a brain arteriovenous malformation	0(0%)		
Moderate or severe ischemic stroke within past 6 months	6 (0.6%)		
Recent major surgery or major trauma within 30 days prior PCI	0(0%)		
Non-deferrable major surgery on DAPT	0(0%)		
**One major criterion**	**351 (36.1%)**	**Two minor criteria**	**278 (28.6%)**
**One major or two minor criteria = HBR: 492 (50.6%)**			
**PRECISE DAPT (PD) SCORE for HBR**			
**Descriptive characteristics for score prediction in whole cohort**			
Hemoglobin (mg/dl)—mean ± SD	13.22 ± 2.05		
Age (years)—mean ± SD	69.77 ± 11.78		
Leukocytes (× 1000/nl)—mean ± SD	9.27 ± 4.83		
Creatinine (mg/dl)—mean ± SD	1.25 ± 1.06		
Prior bleeding—no. (%)	23 (2.4%)		
**PD Score ≥ 25 (HBR): 470 (47.3%)**			

We conducted a retrospective all-comers analysis of 973 NSTEMI patients that underwent percutaneous coronary intervention (PCI) from April 2015 to May 2016. The current DAPT regime was based on clopidogrel for chronic coronary syndrome patients and in combination with oral anticoagulation. Prasugrel and ticagrelor were used in acute coronary syndrome. 22.6% of patients were on triple therapy, 48.2% had 12 months DAPT, 24.1% 6 months DAPT, and 5.1% one to 3 months DAPT. In this cohort, 492 patients (51%) of patients had “high bleeding risk” according to the Academic Research Consortium (ARC) algorithm and 470 (47.3%) according to the PRECISE-DAPT (PD-Score) definition.

The major finding of this study is that, according to the recommendation of the novel European guidelines, more than 50% of NSTEMI patients would be de-escalated to aspirin and clopidogrel for 3 months. This was surprising as limitations of clopidogrel are well known. Hence, more potent P2Y12 inhibitors (prasugrel/ticagrelor) were able to substantially reduce stent thrombosis and re-infarction. In contrast, bleeding was increased. Nevertheless, net clinical benefit was superior for both ticagrelor and prasugrel in comparison to clopidogrel [2, 3].

As mentioned above, detection of “high bleeding risk” is complex. The novel European NSTEMI guidelines emphasized the ARC score. This score includes age, kidney function, non-steroidal anti-inflammatory drugs, and history of stroke among others. Moreover, PD Score is used, depending on hemoglobin and leukocyte levels, age, kidney function, and prior bleeding [4]. However, most parameters predict ischemic events as well. This is in line with a recent evaluation of the ARC “high bleeding risk” definition in post-PCI patients [5]. Moreover, practical usefulness in clinical routine is questionable as not every parameter might be available for every patient. In comparison, the American DAPT guidelines recommend decision for de-escalation and shortening of DAPT down to 6 months on an individual basis integrating clinical judgment, balancing benefit/risk ratio, and patient’s preference without any specific scoring system [6].

In the current analysis, 205 out of the 492 patients with HBR according to ARC definition have an anticipated use of long-term oral anticoagulation as prediction criteria. According to the guidelines for patients with atrial fibrillation, triple therapy is just terminated after one week followed by single antiplatelet treatment combined with oral anticoagulation. Hence, the patients are not considered to receive DAPT for a longer period at all. With the view to DAPT cessation, it might be reasonable to exclude anticoagulation as parameter for HBR determination.

In summary, according to the novel ESC guidelines, more than 50% of NSTEMI patients would receive 3 months of dual antiplatelet therapy with aspirin and clopidogrel. However, it is important to note that this holds potential for an increased ischemic risk, particularly if performed early (< 30 days) after the index event. Large-scale trials are needed to assess the implementation of the novel guidelines to clinical practice and outcome of patients.

## Abbreviations

ACS: Acute coronary syndrome
ARC: Academic Research Consortium
CKD: Chronic kidney disease
DAPT: Dual antiplatelet medication
HBR: High bleeding risk
PCI: Percutaneous coronary intervention
PD: PRECISE-DAPT
